# A Biomathematical Model of Pneumococcal Lung Infection and Antibiotic Treatment in Mice

**DOI:** 10.1371/journal.pone.0156047

**Published:** 2016-05-19

**Authors:** Sibylle Schirm, Peter Ahnert, Sandra Wienhold, Holger Mueller-Redetzky, Geraldine Nouailles-Kursar, Markus Loeffler, Martin Witzenrath, Markus Scholz

**Affiliations:** 1 Institute for Medical Informatics, Statistics and Epidemiology, University of Leipzig, Leipzig, Germany; 2 Department of Internal Medicine/Infectious Diseases and Respiratory Medicine Charité – Universitätsmedizin Berlin, Berlin, Germany; 3 LIFE Research Center of Civilization Diseases, University of Leipzig, Leipzig, Germany; The Hospital for Sick Children and The University of Toronto, CANADA

## Abstract

Pneumonia is considered to be one of the leading causes of death worldwide. The outcome depends on both, proper antibiotic treatment and the effectivity of the immune response of the host. However, due to the complexity of the immunologic cascade initiated during infection, the latter cannot be predicted easily. We construct a biomathematical model of the murine immune response during infection with pneumococcus aiming at predicting the outcome of antibiotic treatment. The model consists of a number of non-linear ordinary differential equations describing dynamics of pneumococcal population, the inflammatory cytokine IL-6, neutrophils and macrophages fighting the infection and destruction of alveolar tissue due to pneumococcus. Equations were derived by translating known biological mechanisms and assuming certain response kinetics. Antibiotic therapy is modelled by a transient depletion of bacteria. Unknown model parameters were determined by fitting the predictions of the model to data sets derived from mice experiments of pneumococcal lung infection with and without antibiotic treatment. Time series of pneumococcal population, debris, neutrophils, activated epithelial cells, macrophages, monocytes and IL-6 serum concentrations were available for this purpose. The antibiotics Ampicillin and Moxifloxacin were considered. Parameter fittings resulted in a good agreement of model and data for all experimental scenarios. Identifiability of parameters is also estimated. The model can be used to predict the performance of alternative schedules of antibiotic treatment. We conclude that we established a biomathematical model of pneumococcal lung infection in mice allowing predictions regarding the outcome of different schedules of antibiotic treatment. We aim at translating the model to the human situation in the near future.

## Introduction

Pneumonia is a high incidence infectious disease and a major cost driver in health care systems. It is one of the leading causes of death worldwide, especially within childhood [1]. Starting as pathogen-driven local inflammation in the lung it might become systemic after destruction of the alveolar-endothelial barrier. This often results in life-threatening organ involvement and sepsis [2, 3].

The outcome of pneumonia infection depends on both, proper antibiotic treatment and the effectivity of the immune response of the host. However, predicting the outcome is a difficult task due to the complexity of the immunologic cascade initiated during infection. Moreover it depends on the pathogen, kind, dosing and timing of antibiotic treatment and individual risk factors [3].

Here, we construct a biomathematical model of murine immune response during pneumonia aiming at predicting the outcome of antibiotic treatment. Focussing on inbred mice data after infection with streptococcus pneumoniae allows us to neglect the impact of different pathogens and a variety of individual parameters influencing outcome of therapies in humans.

Our model consists of a number of non-linear ordinary differential equations describing lung dynamics of pneumococcal population, the inflammatory cytokine IL-6, neutrophils and macrophages fighting the infection and destruction of alveolar tissue due to pneumococcus. Equations were derived by adapting a lung infection model recently proposed by Smith et al. [4]. Corresponding equations are based on the translation of known biological mechanisms of the assumed cascade of immune defence mechanisms consisting of alveolar macrophages fighting the infection first, migrating neutrophils from blood, and finally, migrating monocytes which differentiate to macrophages as the last line of defense [5].

Here, we aim at parametrizing the model on the basis of available mouse data. This requires some adaptations of model equations as well as representation of model compartments by measured biological quantities. Moreover, we included the effects of antibiotic treatment in the equations to explain data during treatment with either Ampicillin or Moxifloxacin.

We study the qualitative behaviour of the model, estimate parameters and discuss their sensitivity in detail. We show how it can be used to predict the outcome of yet untested options of antibiotic therapy demonstrating a way to validate the model on the basis of biologically testable hypotheses.

## Methods

### General Structure of the Model

**Fig 1:**
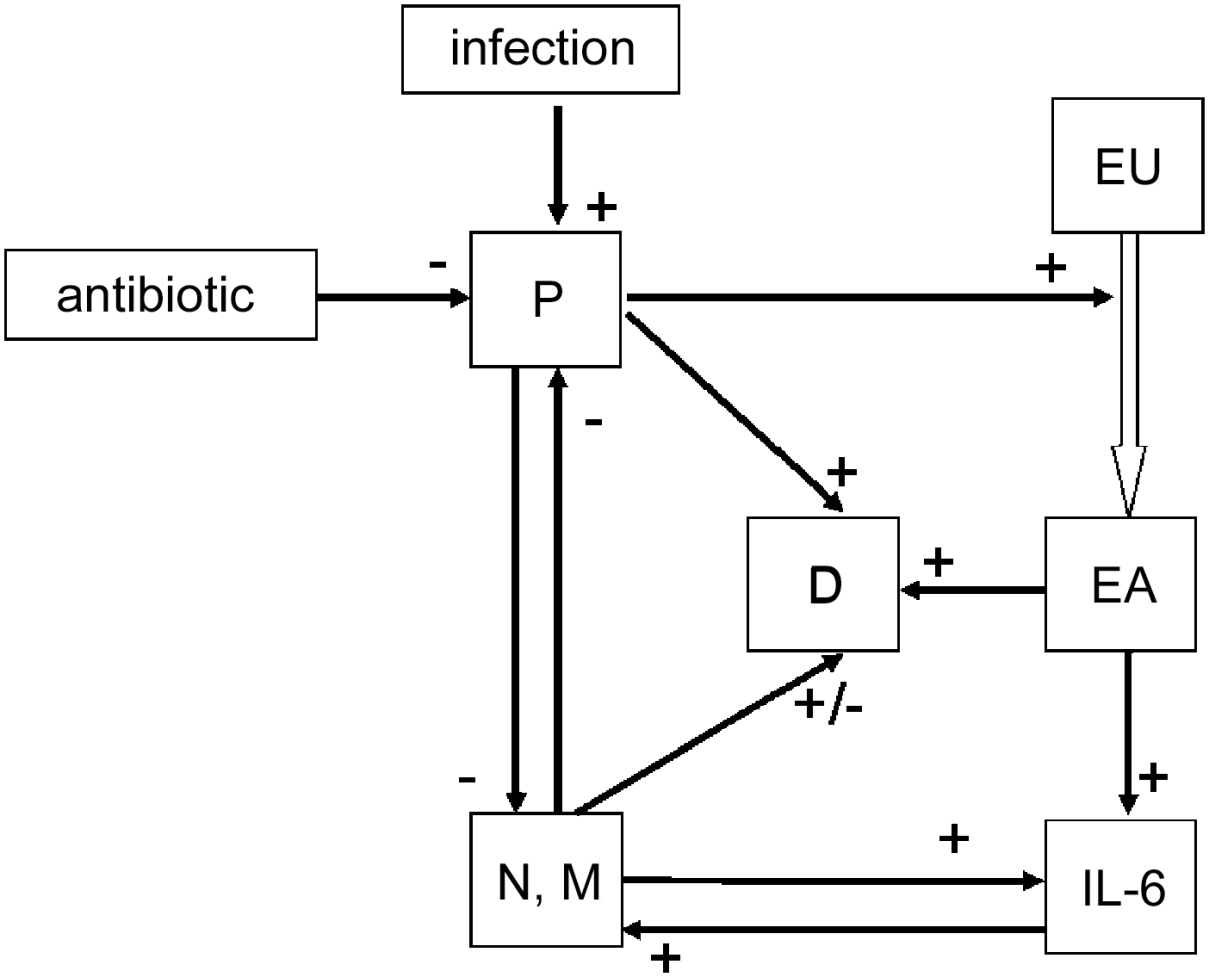
Structure of the model. Compartments: pneumococcal population (P), IL-6, debris (D), unaffected epithelial cells (EU), epithelial cells with pneumococci attached to cell surface receptors (EA) and macrophages/neutrophils (M, N).

We adapted an ordinary differential equation model from Smith et al. [4]. The original model describes the dynamics of pneumococci, neutrophils and monocytes differentiating to macrophages in lung-infected mice. The system is assumed to be regulated by a cytokine response which was modelled phenomenologically. Destruction of the epithelial barrier is also modelled. Here, we aim at parametrizing the model on the basis of experimental mice data of pneumococcus lung infection treated with two antibiotic drugs, namely Moxifloxacin or Ampicillin. This requires some model adaptations including additional compartments and assumptions. We present our model assumptions and equations in the following. A schematic structure of the model is sketched in Fig 1.

### Model equations and assumptions

#### Compartment P: pneumococci

For the bacterial population in the lung alveoli, we assume a logistic growth with rate *k*_*P*_ until saturation *P*_max_ is achieved. The initial value is zero. We always start the simulation with an infectious event. This is modelled by the inhalation function:

$$\begin{array}{c} \text{PneuStart}=\sum_{i=1}^{N}\frac{{\text{dose}}_{\text{Pneu}}^{i}}{t_{\text{Pneu}}}(\text{Hv}\left(t-\tilde{t_{i}}\right)-\text{Hv}\left(t-\tilde{t_{i}}-t_{\text{Pneu}}\right)) \end{array}$$ (1)

where Hv is the Heaviside-function $\text{Hv}=\{\begin{array}{ccc} 0 & : & x<0\\ 1 & : & x\geq 0 \end{array}$, and $\tilde{t_{i}}$ are the time points at which bacteria at dose ${\text{dose}}_{\text{Pneu}}^{i}$ are inhalated. Here, we only consider an initial single infectious event, i.e. $\tilde{t_{i}}=0,N=1$. The inhalation function is multiplied by the factor InfFactor = 0.001. This factor translates applied amount of bacteria to those in 1*μl* broncho-alveolar lung fluid (BALF) after 24 hours. The number of bacteria that actually settles per volume after inhalation of a fixed bacteria dose cannot be determined since no data are available directly after infection (see section data).

Bacteria were eliminated by macrophages and neutrophils. This is modeled by a reaction kinetic where the pneumococcus part is saturated by a Michaelis-Menten kinetic representing the limited killing capacity of an immune cell. This is in contrast to Smith et al. where an unsaturated kinetic was assumed for both, neutrophil and macrophage elimination. Summarizing our considerations result in the following equation.

$$\begin{array}{ccc} \frac{dP}{dt} & = & \text{InfFactor}\cdot \text{PneuStart}+k_{P}\cdot P\left(t\right)\cdot (1-\frac{P(t)}{P_{\text{max}}})\\ & & -k_{\text{PN}}\cdot N\left(t\right)\cdot \frac{P(t)}{n+P(t)}-k_{\text{PM}}\cdot M\left(t\right)\cdot \frac{P(t)}{n+P(t)} \end{array}$$ (2)

We will later add the effect of antibiotic treatment to this equation.

#### Compartment EU: unaffected epithelial/endothelial cells

The barrier between the lung and the circulation is formed by epithelial and endothelial cells (endoepithelial barrier). Here, we do not distinguish between epithelial and endothelial cells and refer to *epithelial cells* in the following according to Smith et al. A major disease mechanism of pneumococci is the destruction of this barrier increasing the permeability of the barrier eventually resulting in a systemic infection. Therefore, we model the compartment of unaffected epithelial cells in the following way.

In the absence of pneumococci, the epithelium is in steady-state. During infection, epithelial cells transform to affected epithelial cells according to an unsaturated reaction kinetic of first order with rate parameter *k*_EUP_. Thus

$$\begin{array}{c} \frac{dEU(t)}{dt}=k_{\text{EU}}\cdot (1-\frac{EU(t)}{EU_{0}})-k_{\text{EUP}}\cdot P\left(t\right)\cdot EU(t) \end{array}$$ (3)

i.e. in contrast to Smith et al. [4], our equations return to steady state after elimination of pneumococci [6].

#### Compartment EA: affected epithelial cells

The compartment EA describes epithelial cells affected by pneumococci. The influx of this compartment equals the second term of Eq (3). We assume that the cells are destroyed in a first order kinetic transforming the cells into debris. A constant rate *d*_*E*_ is assumed for this process.

$$\begin{array}{c} \frac{dEA(t)}{dt}=k_{\text{EA}}\cdot P\left(t\right)\cdot EU(t)-d_{E}\cdot EA(t) \end{array}$$ (4)

Since epithelial cells under attack produce pro-inflammatory cytokines [7], we assume that EA regulates the cytokine production considered in the next equation.

#### Compartment C: cytokine IL-6

In analogy to [4], cytokines were produced by affected epithelial cells and by macrophages. While in [4] the compartment C addresses the cytokines TNF-*α* and IL-1, we consider it as a representative of IL-6 available in our data. We assume a production by affected epithelial cells at rate *k*_CEA_ and by macrophages at rate *k*_CMA_. Further, we assume a constant baseline production *P*_C_. In steady state there is a cytokine level of *C*_0_.

$$\begin{array}{c} \frac{dC(t)}{dt}=k_{\text{CEA}}\cdot EA\left(t\right)+k_{\text{CMA}}\cdot M\left(t\right)\cdot P\left(t\right)-d_{C}\cdot C\left(t\right)+P_{C} \end{array}$$ (5)

with the (implicit) steady state condition

$$\begin{array}{c} P_{C}=d_{C}\cdot C_{0} \end{array}$$ (6)

#### Compartment N: neutrophils

Neutrophils were assumed to be positively correlated with the cytokine IL-6 with factor *k*_NC_, limited by the maximum number *N*_max_ [4, 8]. In analogy to [4], they were reduced by bacteria at rate *d*_NP_ or by natural cell death at rate *d*_*N*_. In steady state we assume a neutrophil count of *N*_0_, realised by a baseline recruitment *P*_N_.

$$\begin{array}{ccc} \frac{dN(t)}{dt} & = & k_{\text{NC}}\cdot C\left(t\right)\cdot (1-\frac{N(t)}{N_{\text{max}}})\\ & & -d_{\text{NP}}\cdot N\left(t\right)\cdot P\left(t\right)-d_{N}\cdot N\left(t\right)+P_{N} \end{array}$$ (7)

where

$$\begin{array}{c} P_{N}=d_{N}\cdot N_{0}-k_{\text{NC}}\cdot C_{0}\cdot (1-\frac{N_{0}}{N_{\text{max}}}) \end{array}$$ (8)

#### Compartment D: debris

In analogy to [4], apoptotic neutrophils, eliminated bacteria and affected epithelial cells contribute to the increase of debris at rates *k*_DN_, *k*_DNP_ and *k*_DEA_. Debris is cleared by macrophages at rate *k*_MAD_, and by other processes with rate *d*_*D*_. In the data available for our modelling [9], debris is measured in terms of a semi-quantitative histological score. Therefore, we adapted the parameters accordingly.

$$\begin{array}{ccc} \frac{dD(t)}{dt} & = & k_{\text{DNP}}\cdot N\left(t\right)\cdot P\left(t\right)+k_{\text{DN}}\cdot N\left(t\right)+k_{\text{DEA}}\cdot EA\left(t\right)\\ & & -k_{\text{MAD}}\cdot M\left(t\right)\cdot D\left(t\right)-d_{D}\cdot D\left(t\right) \end{array}$$ (9)

with

$$\begin{array}{c} d_{D}=\frac{k_{\text{DN}}\cdot N_{0}-k_{\text{MAD}}\cdot M_{0}\cdot D_{0}}{D_{0}} \end{array}$$ (10)

In contrast to [4], *D*_0_ is set to 1, because the sham+solvent group data from [9] showed a mean score of 1.

#### Compartment M: macrophages

In steady state, there is a pool of alveolar macrophages fighting the infection first [5]. Additionally, monocyte to macrophage differentiation was promoted by IL-6 [10]. In our model, we do not distinguish between alveolar macrophages and migrated monocyte derived macrophages.

$$\begin{array}{c} \frac{dM(t)}{dt}=\frac{k_{\text{MAC}}\cdot k_{M}\cdot C\left(t\right)}{C\left(t\right)+k_{M}}-d_{M}\cdot M\left(t\right)+P_{M} \end{array}$$ (11)

with steady state condition

$$\begin{array}{c} P_{M}=d_{M}\cdot M_{0}-\frac{k_{\text{MAC}}\cdot k_{M}\cdot C_{0}}{C_{0}+k_{M}} \end{array}$$ (12)

### Modelling antibiotic treatment

To describe the effect of antibiotic therapy, we developed an injection function for the different antibiotic drugs. The antibiotics injection function ABIO^inj^(*t*) can be written as a sum of pulse functions

$$\begin{array}{cc} {\text{ABIO}}^{\text{inj}}\left(t\right)= & \;\frac{{\text{dose}}_{\text{ABIO}}\cdot k_{\text{ABIO}}}{t_{\text{ABIO}}}\cdot \sum_{i=1}^{N}(\text{Hv}\left(t-\tilde{t_{i}}\right)-\text{Hv}\left(t-\tilde{t_{i}}-t_{\text{ABIO}}\right)) \end{array}$$ (13)

where ABIO ∈ {Amp, Mox}, and $\tilde{t_{i}}$ are the time points at which antibiotics at dose dose_ABIO_ were administered. In [9], 0.02 mg/g Ampicillin or 0.1 mg/g Moxifloxacin were applied. *k*_Amp_ and *k*_Mox_ are amplification factors describing the antibiotic effect of the specific drugs. The duration of injection (*t*_ABIO_) is set to 0.1 hour. The applied dose is normalised to this injection time by the factor 1/*t*_ABIO_.

### Modelling pharmacokinetics of antibiotics

We assume that antibiotic treatment acts with some time delay modelled by two delay compartments, where $C_{\text{ABIO}}^{\left(i\right)}\left(t\right)$ is the concentration of the antibiotic drug in the compartment *i*:

$$\begin{array}{c} \frac{dC_{\text{ABIO}}^{\left(i\right)}\left(t\right)}{dt}=C_{\text{ABIO}\_\text{out}}^{\left(i-1\right)}\left(t\right)-k_{\text{ABIO}}^{\text{Delay}}\cdot C_{\text{ABIO}}^{\left(i\right)}\left(t\right)\;i=1,2 \end{array}$$ (14)

with the settings

$$\begin{array}{c} C_{\text{ABIO}\_\text{out}}^{\left(0\right)}\left(t\right)={\text{ABIO}}^{\text{inj}}\left(t\right) \end{array}$$

and

$$\begin{array}{c} C_{\text{ABIO}\_\text{out}}^{\left(i\right)}\left(t\right)=k_{\text{ABIO}}^{\text{Delay}}\cdot C_{\text{ABIO}}^{\left(i\right)}\left(t\right)\;i=1,2 \end{array}$$

**Fig 2:**
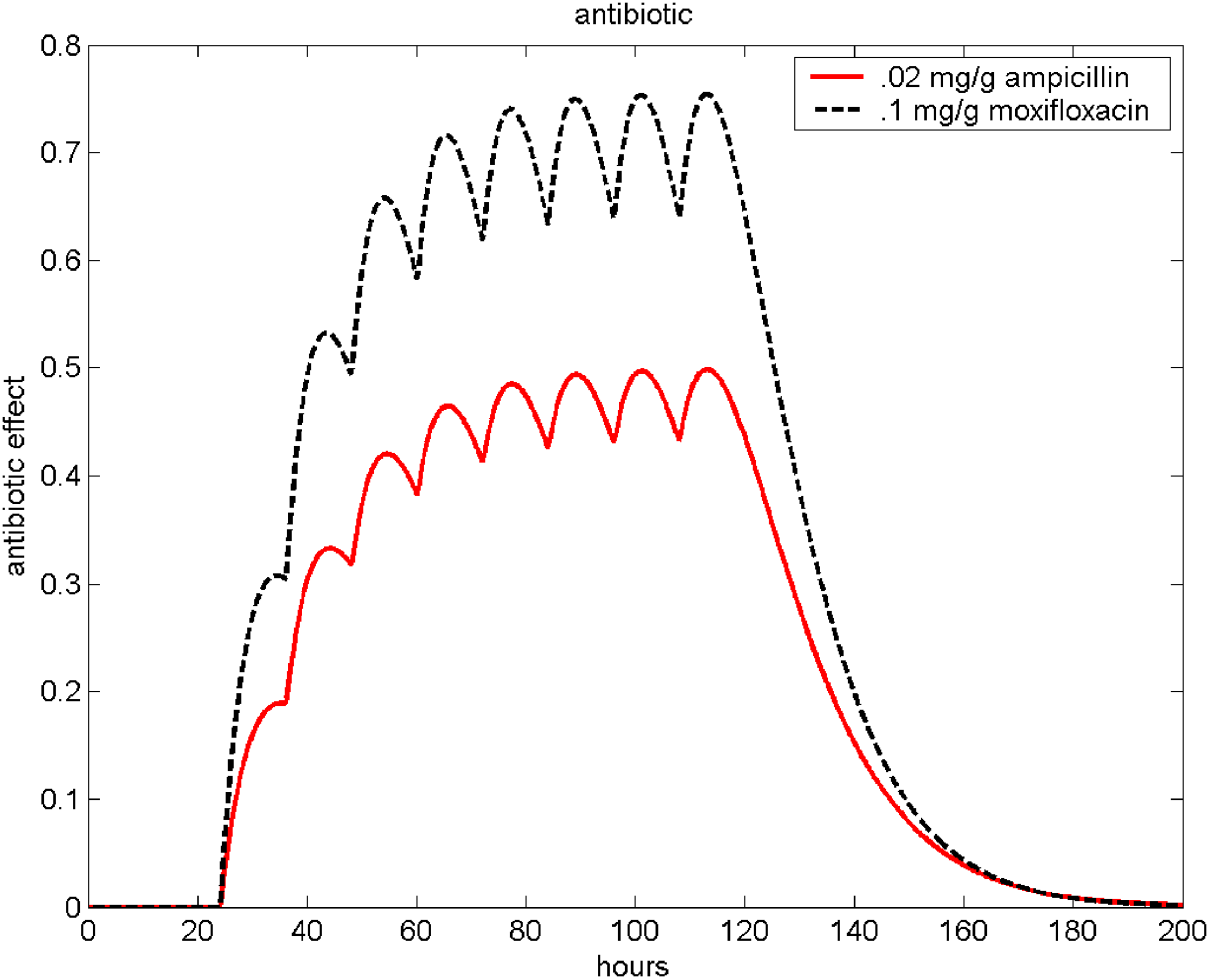
Antibiotic therapy. The resulting antibiotic effect function *A*(*t*) of Ampicillin (0.02mg/g) and Moxifloxacin (0.1 mg/g) is shown. Antibiotics were given every 12 hours, starting 24 hours after infection.

$C_{\text{ABIO}\_\text{out}}^{\left(2\right)}\left(t\right)$ enters the compartment of antibiotic effect on pneumococci:

$$\begin{array}{c} \frac{dA(t)}{dt}=C_{\text{ABIO}\_\text{out}}^{\left(2\right)}\left(t\right)-d_{\text{ABIO}}\cdot A\left(t\right) \end{array}$$ (15)

where *A*(*t*) corresponds to the antibiotic effect and *d*_ABIO_ is the elimination rate. The antibiotic effect *A*(*t*) of repetitive Moxifloxacin (0.1mg/g) and Ampicillin (0.02mg/g) applications is illustrated in Fig 2. Adding antibiotic therapy, Eq (2) extends to

$$\begin{array}{ccc} \frac{dP}{dt} & = & \text{InfFactor}\cdot \text{PneuStart}+k_{P}\cdot P\left(t\right)\cdot (1-\frac{P(t)}{P_{\text{max}}})\\ & & -k_{\text{PN}}\cdot N\left(t\right)\cdot \frac{P(t)}{n+P(t)}-k_{\text{PM}}\cdot M\left(t\right)\cdot \frac{P(t)}{n+P(t)}-A\left(t\right)\cdot P\left(t\right) \end{array}$$ (16)

### Numerical Methods for Simulation

Simulations were performed with MATLAB 7.5.0.342 (R2007b) using the SIMULINK toolbox (The MathWorks Inc., Natick, MA, USA). Numerical solutions of the equation system are obtained using the variable step solver from Adams and Bashford (ode113, SIMULINK toolbox).

### Data

For our model, we used the data published in [9], which were originally collected for comparing the anti-inflammatory effect of Moxifloxacin and Ampicillin. Data comprise time series of pneumococci (measured as colony-forming units), neutrophils, and macrophages in BALF (no differentiation between alveolar macrophages and those derived from immigrated monocytes possible), IL-6 measured in BALF, debris assessed by a histological score of the lung tissue and permeability (measured by the ratio of BALF and murine serum albumine).

#### Murine pneumonia

Animal experiments were approved by institutional and governmental (LaGeSo Berlin) authorities and performed according to the Helsinki convention for the use and care of animals.

Female C57Bl/N mice (8–10 weeks old) were anaesthetized by i.p. ketamine (1.6 mg) and xylazine (0.5 mg). Next, mice were transnasally inoculated with 5 ⋅ 10^6^ cfu S. pneumoniae serotype 3 (strain PN36, NCTC7978) in 20 *μl* PBS. At the respective experimental endpoints, animals were deeply anaesthetized, subjected to mechanical ventilation after tracheotomy and exsanguinated by puncture of the vena cava inferior following median laparotomy.

#### Leukocyte differentiation in bronchoalveolar lavage fluid and blood

Lungs were flushed blood free via the pulmonary artery and lavaged two times with 800 *μl* PBS each. Leukocytes were isolated from bronchoalveolar lavage fluid (BALF) by centrifugation, pooled, counted and differentiated by flow cytometry (FacsCalibur, BD, Heidelberg, Germany) according to their forward and side scatter properties, and by CD45, F4/80, Gr-1 staining (BD, Heidelberg, Germany).

Leukocytes were quantified from whole blood using Trucount tubes and differentiated by forward and side scatter properties, and by their CD45, Gr-1 staining properties (BD, Heidelberg, Germany).

#### Cytokine Multiplex Assays

Cytokines were measured in BALF and blood plasma by multiplex assay technique according to the manufacturer’s instructions (Bio-Rad, Hercules, CA, USA)

To compare model and data, we used the following analogies of measured features and modeled compartments (see Table 1).

**Table 1:** Data and corresponding model compartments.

measured quantity	explanation	model compartment
CFU	pneumococci in BALF	P (pneumococci)
IL-6	IL-6 measured in BALF	C (cytokine)
Lung infection	histological score of epithelial	D (debris)
score	damage, semi-quantitative	
PMN	neutrophils in BALF	N (neutrophils)
AM	macrophages in BALF	M (macrophages)
Permeability	proxied by ratio BALF/serum albumine	EA (affected epithelial cells)

Steady state values for *N*, *M* and *EA* are calculated by the geometric means of the sham+solvent group at time points *t*_1_ = 72, *t*_2_ = 120. We use the mean of the time point 120 as the steady state value of IL-6. The median and minimum values of lung infection score equals 1 at all timepoints, therefore we set *D*_0_ = 1 (see Table 2).

**Table 2:** Initial values.

parameter	meaning	value	
*P*_0_	initial value of bacteria	0.00E+00	set
*EU*_0_	initial value of epithelial cells	1.00E+02	set
*EU*_nor_	normal value of epithelial cells	1.00E+02	set
*EA*_0_	initial value of activated epithelial cells	0.00E+00	set
*C*_0_	initial value of IL-6	3.90E-01	set
*D*_0_	initial value of debris	1.00E+00	set
*M*_0_	initial number of macrophages	3.61E+04	set
*N*_0_	initial number of neutrophils	2.09E+02	set

### Estimation of Parameters

To determine optimal parameter settings, we used a pointwise optimisation function for fitting model predictions to the data. Let (*t*_*i*_, *x*_*i*_), (*i* = 1, …, *N*) be the time points *t*_*i*_ with corresponding normalised measurements *x*_*i*_ and let *f*(*t*; **k**) the solution of the differential equation system. We solve the extremal value problem

$$\begin{array}{c} \sum_{i=1}^{n}|x_{i}-f\left(t_{i};k\right)|\to \text{minimise}\;\text{with}\;\text{respect}\;\text{to}\;k \end{array}$$ (17)

The left hand side is refered to as the fitness function. At this, fitness functions of different scenarios were added. To find optimal parameter settings, evolutionary algorithms with self-adapting mutation step size were used [11, 12]. These are non-deterministic optimisation algorithms based on mutation, realisation and survival of the fittest.

To our experiences, in order to fit differential equations-based models to data, evolutionary strategies outperform for example gradient methods since fewer computationally expensive evaluations of the fitness function are required. Moreover, there is a higher chance that a global optimum is found. Finally, it has been estimated that evolutionary strategies achieve the optimum more quickly especially if the number of parameters is high [11], p.126, figure 31. We successfully fitted a number of highly complex, multi-parametric models applying evolutionary strategies (see [13–18]).

Here, we used an evolutionary strategy with one parent having three children in each generation (see [11, 12] for further details). A list of the estimated parameters can be found in Table 3.

**Table 3:** Parameter values of the model.

parameter	meaning	value	
*k*_P_	pneumococcal population growth rate	7.90E-01	fitted
*P*_max_	maximal number of bacterials	7.50E+03	fitted
*k*_PN_	bacterial clearance by neutrophils	5.98E-04	fitted
*k*_PM_	bacterial clearance by macrophages	6.67E-03	fitted
*k*_EU_	epithelial cells growth rate	9.39E-02	fitted
*k*_EUP_	bacterial attachment to epithelial cells	4.33E-07	fitted
*k*_EA_	epitelial cells activation rate	6.79E-07	fitted
*d*_E_	epithelial cell degradation rate	1.52E-02	fitted
*k*_CEA_	cytokine production by epithelial cells	3.13E+03	fitted
*k*_CMA_	cytokine production by macrophages	1.09E-04	fitted
*N*_max_	maximal number of neutrophils	1.80E+06	set
*k*_NC_	neutrophil recruitment rate	1.33E+03	fitted
*d*_NP_	bacterial-induced neutrophil death rate	1.05E-09	fitted
*k*_DNP_	debris from bacterial-induced neutrophil death	4.26E-12	fitted
*k*_DN_	debris from neutrophil death	2.42E-08	fitted
*k*_DEA_	debris from epithelial cell death	3.81E-04	fitted
*d*_D_	debris degradation rate	4.62E-06	fitted
*k*_MAD_	removal of debris by macrophages	1.21E-11	fitted
*k*_MAC_	macrophage recruitment rate by IL-6	6.33E+02	fitted
*d*_M_	macrophage degradation rate	5.93E-01	fitted
*n*	maximum number of bacteria per M, N	1.00E+02	set
*d*_C_	IL-6 clearance rate	2.01E+02	fitted
*d*_N_	neutrophil degradation rate	2.48E+00	fitted
*k*_M_	constant	8.30E+01	fitted
P_N_	neutrophile production	5.18E-05	set
P_C_	cytokine production	7.83E+01	set
P_M_	macrophage production	2.12E+04	set
*t*_Pneu_	inhalation time	1.67E-02	set
InfFactor	rate of bacterials colonizing after inhalation	1.00E-03	set
*t*_ABIO_	injection time for antibioticum	1.00E-01	set
*k*_Amp_	antibiotic effect factor of Ampicillin	2.00E+03	fitted
*k*_Mox_	antibiotic effect factor of Moxifloxacin	6.00E+02	fitted
$k_{\text{Amp}}^{\text{Delay}}$	delay of antibiotic effect	9.00E-02	fitted
$k_{\text{Mox}}^{\text{Delay}}$	delay of antibiotic effect	9.76E-02	fitted
*d*_Amp_	clearance of antibioticum	7.00E+00	fitted
*d*_Mox_	clearance of antibioticum	7.00E+00	fitted

## Results

### Qualitative model behaviour

At first, we study the qualitative behaviour of our differential equation system. It can be shown that the initial condition *P* = *P*_0_, *N* = *N*_0_, *M* = *M*_0_, *C* = *C*_0_, *EA* = *EA*_0_, *EU* = *EU*_0_, *D* = *D*_0_ represents a stable steady-state of the equations, i.e. the system returns to it after cure.

*Proof*. Compartment *D* is not coupled, and consequently, has no impact on stability. Considering the following order of state variables: *P*, *EU*, *EA*, *C*, *N*, *M*, the Jacobian of the system at the steady state reads as follows:

$$\begin{array}{c} J=(\begin{array}{cccccc} k_{P}-\frac{k_{\text{PN}}\cdot N_{0}+k_{\text{PM}}\cdot M_{0}}{n} & 0 & 0 & 0 & 0 & 0\\ -k_{\text{EUP}}\cdot EU_{0} & -\frac{k_{\text{EU}}}{EU_{0}} & 0 & 0 & 0 & 0\\ k_{\text{EA}}\cdot EU_{0} & 0 & -d_{E} & 0 & 0 & 0\\ k_{\text{CMA}}\cdot M_{0} & 0 & k_{\text{CEA}} & -d_{C} & 0 & 0\\ -d_{\text{NP}}\cdot N_{0} & 0 & 0 & k_{\text{NC}}\cdot \left(1-\frac{N_{0}}{N_{\text{max}}}\right) & -\frac{k_{\text{NC}}\cdot C_{0}}{N_{\text{max}}}-d_{N} & 0\\ 0 & 0 & 0 & \frac{k_{\text{MAC}}\cdot k_{M}^{2}}{{\left(k_{M}+C_{0}\right)}^{2}} & 0 & -d_{M} \end{array}) \end{array}$$

Thus, eigenvalues of **J** are $k_{P}-\frac{k_{\text{PN}}N_{0}+k_{\text{PM}}M_{0}}{n}=-1.6201$, $-\frac{k_{\text{EU}}}{EU_{0}}=-9.3868\cdot 10^{-4}$, −*d*_*E*_ = −0.0152, −*d*_*C*_ = −200.8505, $-\frac{k_{\text{NC}}\cdot C_{0}}{N_{\text{max}}}-d_{N}=-2.4770$, and −*d*_*M*_ = −0.5934. Since all eigenvalues are strictly negative, the steady-state is locally asymptotically stable.

**Fig 3:**
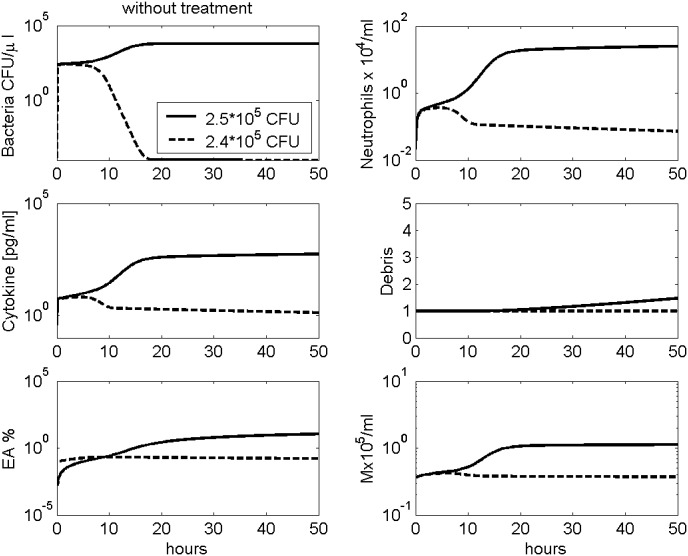
Modelling different initial doses of pneumococci without treatment. Time series of pneumococcal population, neutrophils, IL-6, debris, EA and macrophages in BALF are presented. The solid black curves represent simulation results with an initial bacterial load of 2.5 ⋅ 10^5^, the dashed lines with 2.4 ⋅ 10^5^. As one can see, the smaller initial dose is successfully removed by the immune system, while larger doses result in disseminated infection.

Depending on the initial bacterial load, simulation shows different results: As a major requirement for biological plausibility, lower initial doses of pneumococci were successfully removed by the immune system. However, initial bacterial load above 2.5 ⋅ 10^5^ results in a disseminated infection (see Fig 3).

Dropping the debris variable which is continuously increasing during this process (eventually resulting in the death of animals), the limit represents another locally stable steady-state with respect to the other state variables. Numerically obtained values for this steady-state and corresponding stability analysis can be found in the supplement (see S1 Appendix).

**Fig 4:**
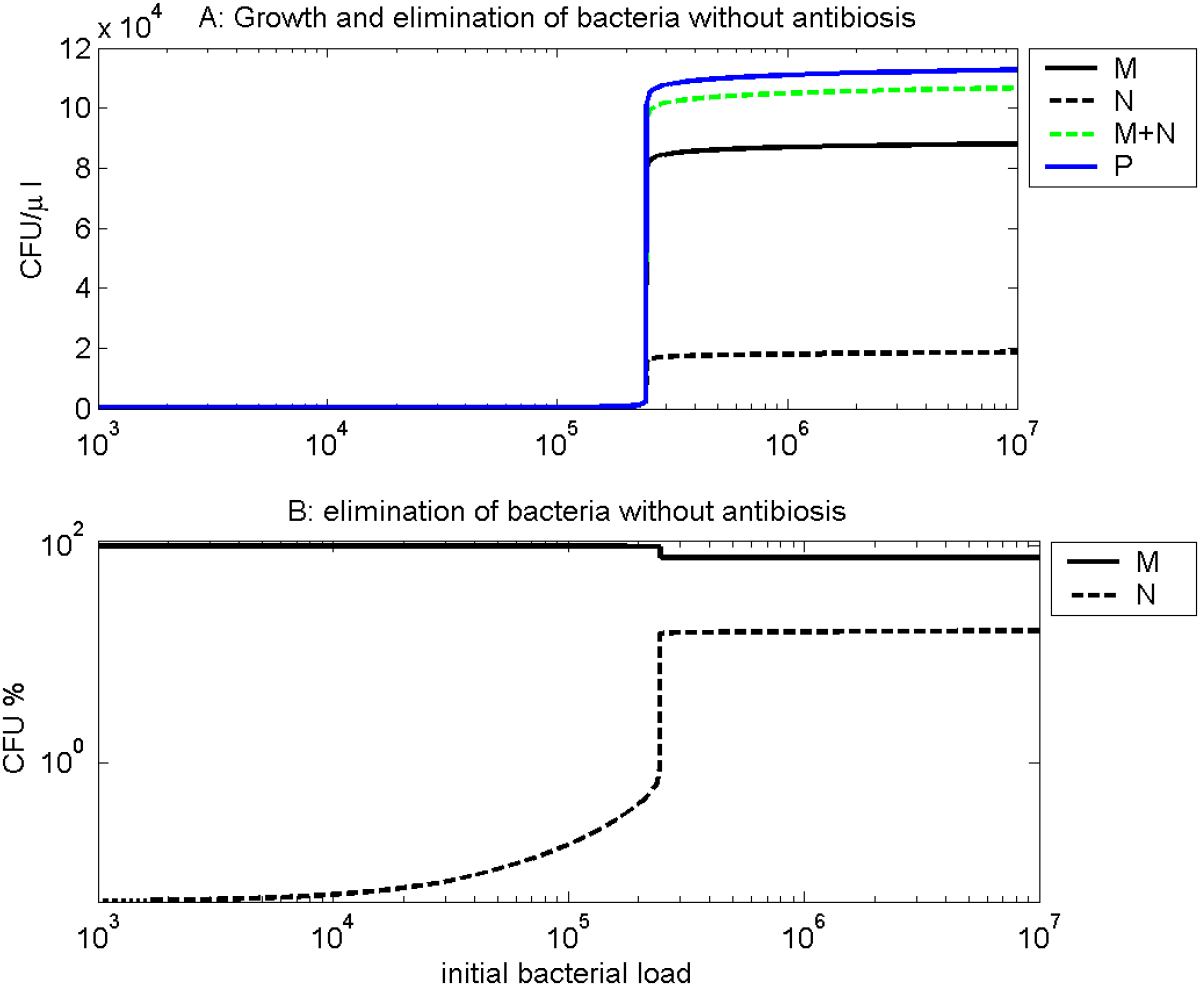
Bacterial growth and elimination by the immune system. We simulated different initial dosages of bacteria without antibiotic treatment. A: In dependance on the inhalated dose (x axis), we calculate the total amount of bacteria produced over a period of 120 hours per *μl* of BALF (blue line), the total amount of bacteria eliminated by neutrophils over this period (black dashed line), and the total amount of bacteria eliminated by macrophages over the same period (black solid line). The green dashed line represents the sum of bacteria eliminated by neutrophils and macrophages over the period. As one can see, immune system is unable to remove all bacteria if the initial dose exceeds about 2.5 ⋅ 10^5^. B: In dependance on the initial dose, the percentage of bacteria eliminated by neutrophils respectively macrophages over a period of 120 hours is depicted. After infection with lower doses, 100% of bacteria were eliminated by macrophages (solid line) over a period of 120 hours. For higher doses, neutrophils are also involved (dashed line).

Fig 4A shows the growth of pneumococci and their elimination by neutrophils and macrophages. We calculate the total amount of bacteria growing over a period of 120 hours per *μl* of BALF, and the total amount of bacteria eliminated by neutrophils respectively by macrophages over this period. Low numbers of bacteria were eliminated successfully by existing (alveolar) macrophages, while a higher starting dose needs the commitment of neutrophils. For sufficiently high initial doses, the bacteria can not be controlled by the immune system. Fig 4B shows the percental contribution of neutrophils and macrophages to elimination of bacteria for different initial doses. For macrophages it is 100% for low doses. Neutrophils contribute only at sufficiently high doses. This is in agreement with the biological understanding of the process.

### Parameter sensitivity

**Fig 5:**
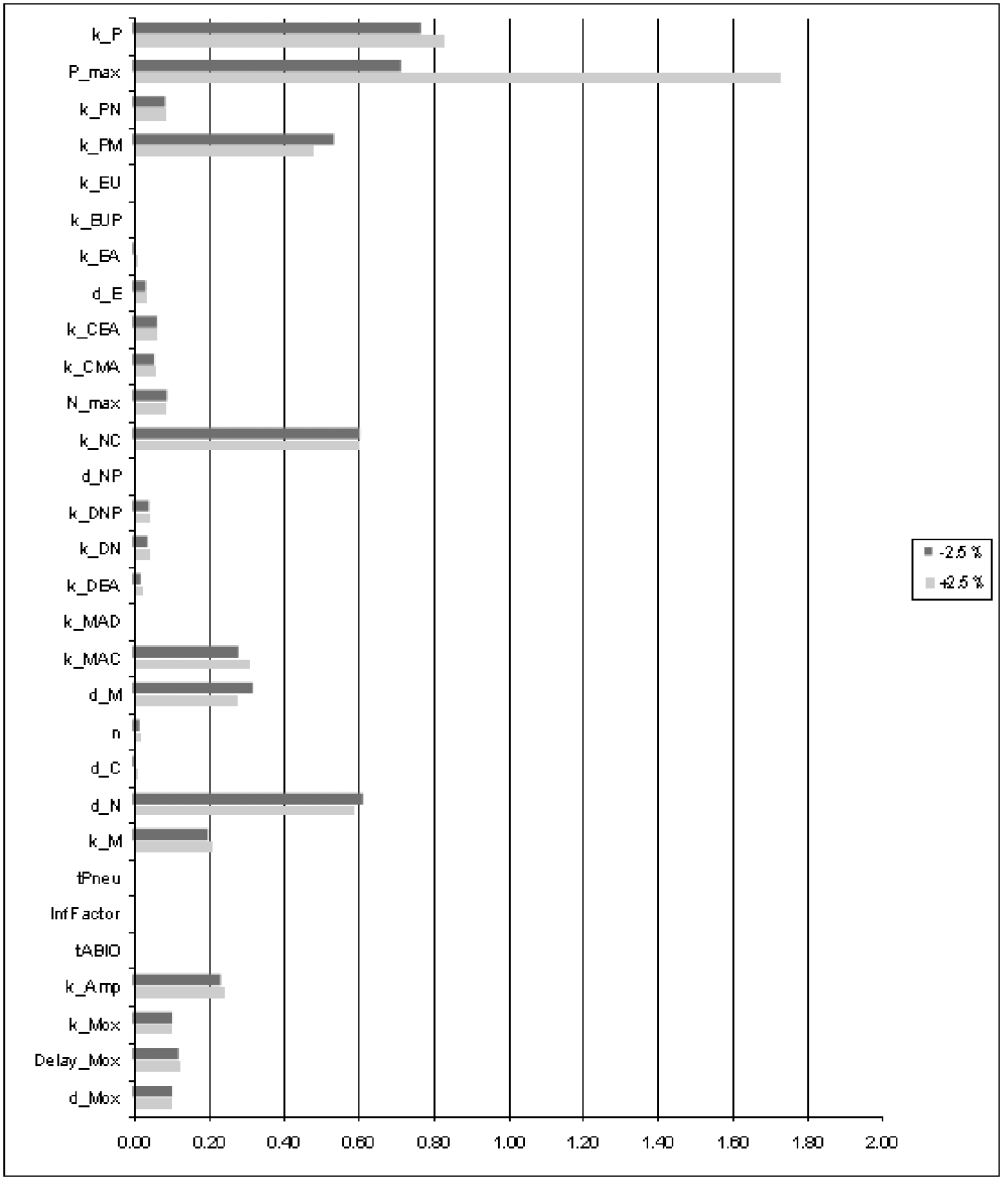
Sensitivity of model parameters. Single parameter values were changed by ±2.5% while the other parameters were kept constant. Corresponding percental deterioration of the fitness function Eq (17) was evaluated as a measure of sensitivity of the considered parameter. Longer bars correspond to more sensitive parameters, i.e. better identifiability.

We performed an extensive sensitivity analysis in order to determine the identifiability of all model parameters. This is achieved by estimating the deterioration in fitness after changing the parameters by +/- 2.5% (see Fig 5). The parameters maximum amount of pneumococci (*P*_max_), pneumococcal population growth rate (*k*_P_), bacterial clearance by macrophages (*k*_PM_), the recruitment rates of macrophages and neutrophils (*k*_MAC_, *k*_NC_) and the degradation rates of macrophages and neutrophils (*d*_M_, *d*_N_) are most sensitive.

### Comparison: model and data

**Fig 6:**
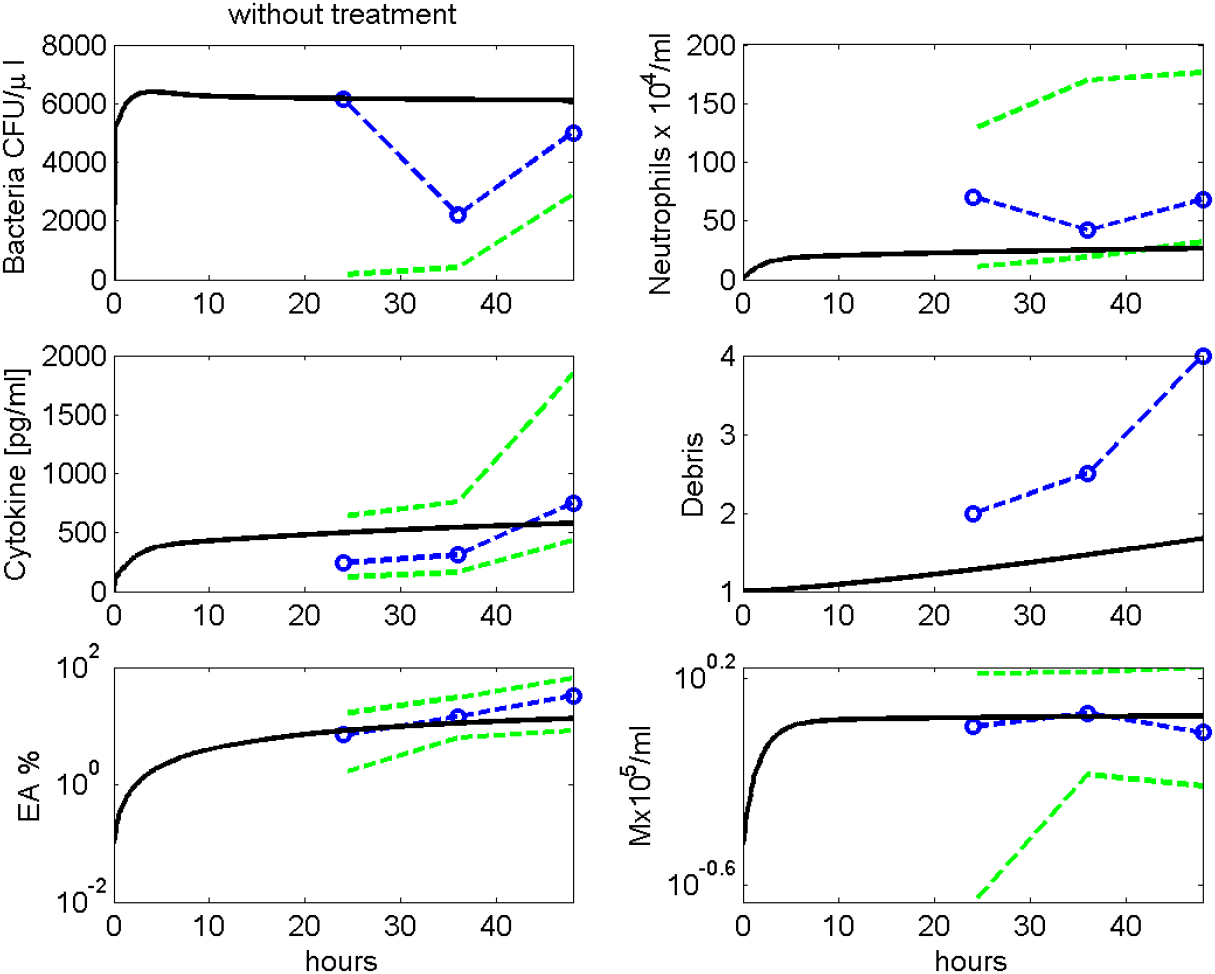
Modelling infection without treatment. We compare results of model simulation with data from [9] without antibiotic treatment. Time series of pneumococcal population, neutrophils, IL-6, debris, EU, EA and macrophages in BALF are displayed. Solid black curve represents simulation results. Circles represent data points. The green dashed lines represent minimal and maximal observed values.

Parameter estimates resulted in a good agreement of model and data for the available scenarios. In Fig 6, the simulated time courses of pneumococcal population, neutrophils, IL-6, debris, EU, EA and macrophages in BALF after infection with 5 ⋅ 10^6^ bacteria without treatment are presented and compared with corresponding data from [9]. For untreated mice, simulation shows that bacteria remain on an almost constant level for more than 48 hours. This is in rough agreement with the data. The apparent drop of bacteria at 36 hours is not statistically significant (p = 0.26, Kruskal-Wallis test).

**Fig 7:**
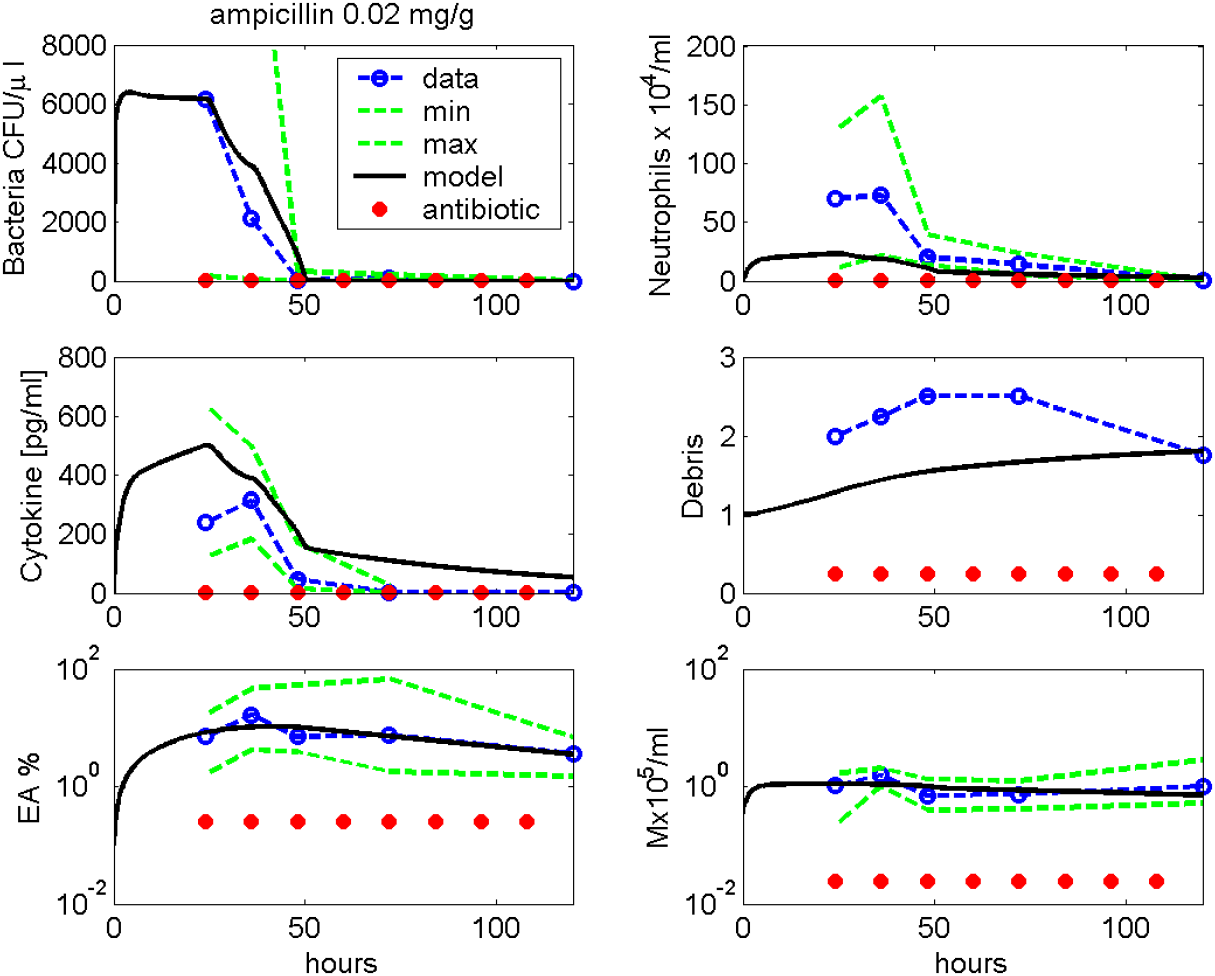
Ampicillin treatment. We compare results of model simulation with data from [9] under Ampicillin treatment. Therapy starts 24 hours after infection and is continued every 12h. Time series of pneumococcal population, neutrophils, IL-6, debris, EU, EA and macrophages in BALF are displayed. Solid black curve represents simulation results. Circles represent data points. The green dashed lines represent minimal and maximal observed values.

**Fig 8:**
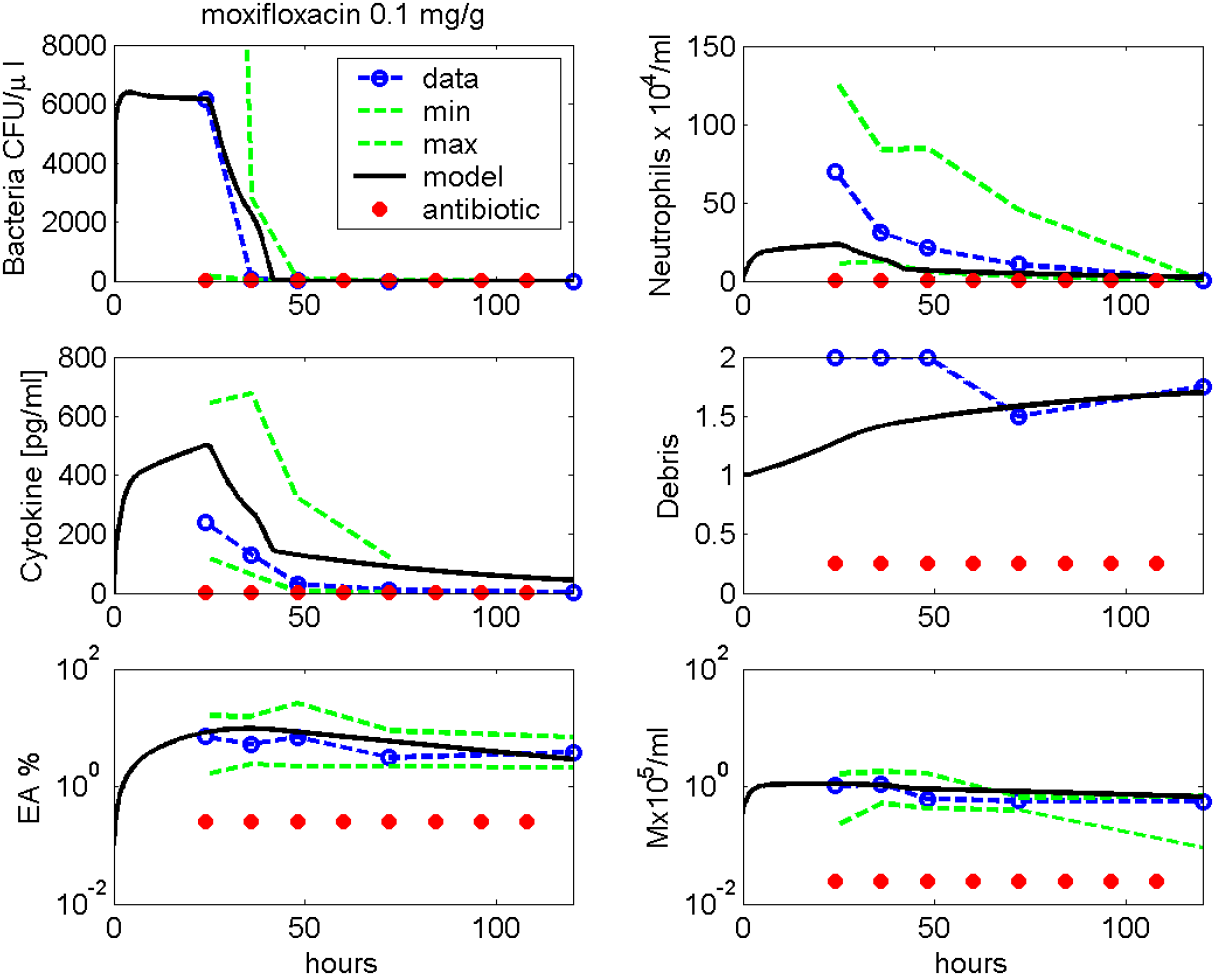
Moxifloxacin treatment. We compare results of model simulation with data from [9] under Moxifloxacin treatment. Therapy starts 24 hours after infection and is continued every 12h. Time series of pneumococcal population, neutrophils, IL-6, debris, EU, EA and macrophages in BALF are displayed. Solid black curve represents simulation results. Circles represent data points. The green dashed lines represent minimal and maximal observed values.

Figs 7 and 8 show comparisons of model simulations and data from infections with 5 ⋅ 10^6^ Streptococcus pneumoniae treated with either 0.02 mg/g Ampicillin or 0.1 mg/g Moxifloxacin (data taken from [9]). The antibiotic therapies started 24 hours after infection, and the antibiotics were given every 12 hours. However, according to our model results, bacteria are eradicated already at 72 hours while numbers of immune cells are still higher than in steady-state.

### Prediction

**Fig 9:**
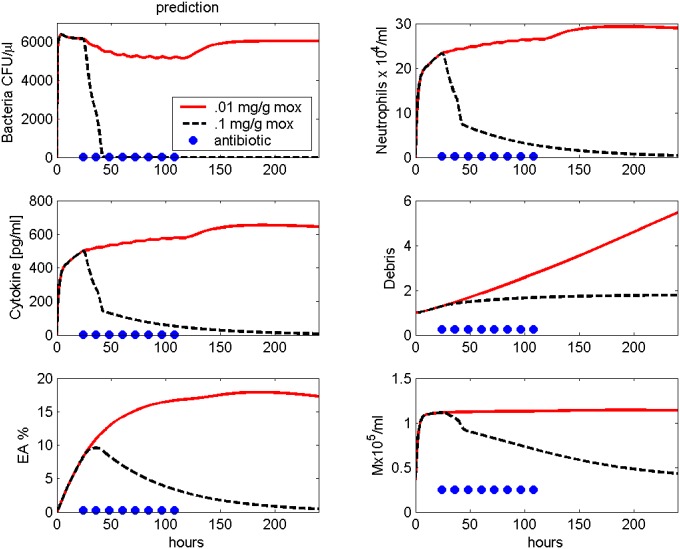
Prediction: Moxifloxacin treatment. Treatment with different doses of Moxifloxacin after infection with 5 ⋅ 10^6^ Streptococcus pneumoniae are simulated. Moxifloxacin is given every 12 hours starting 24 hours after infection. Time series of pneumococcal population, neutrophils, IL-6, debris, EU, EA and macrophages in BALF are presented. Solid red curves represent the predicted time course with lower dosed Moxifloxacin (0.01 mg/g). The black dashed line represents the simulation results of the higher dose (0.1 mg/g). Circles represent time points of antibiotic treatment.

After calibration, the model can be used to predict new therapy schedules. We simulated an infection with 5 ⋅ 10^6^ bacteria and Moxifloxacin treatment every 12 hours starting 24 hours after infection with lower dosage (0.01 mg/g) in comparison to [9]. While with a dose of 0.1 mg/g the bacteria can be eliminated quickly, the therapy with 0.01 mg/g Moxifloxacin is unsuccessful (see Fig 9).

**Fig 10:**
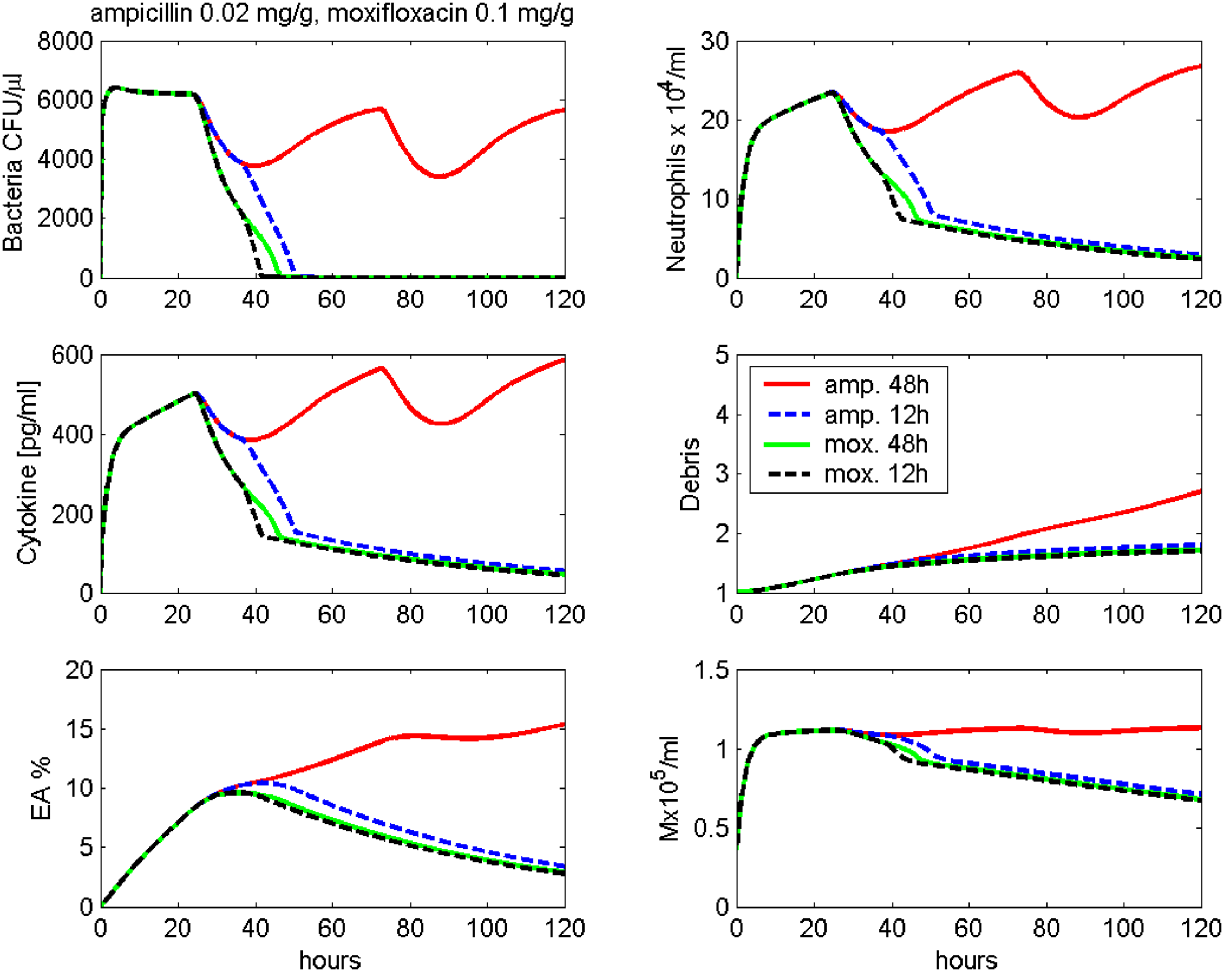
Prediction: Different schedules of antibiotic treatment. Treatment with different schedules of Ampicillin or Moxifloxacin after infection with 5 ⋅ 10^6^ Streptococcus pneumoniae are simulated. Time series of pneumococcal population, neutrophils, IL-6, debris, EU, EA and macrophages in BALF are presented. Ampicillin 0.02 mg/g is given every 12 hours starting 24 hours after infection (dashed blue line) or every 48 hours starting 24 hours after infection (red line). Moxifloxacin 0.1 mg/g is given every 12 hours starting 24 hours after infection (dashed black line) or every 48 hours starting 24 hours after infection (green line).

Next, we study different time schedules with the same dosage. In Fig 10, we simulate 0.02 mg/g Ampicillin treatment every 12 hours and every 48 hours, starting 24 hours after infection. While the denser treatment can eliminate the bacteria, the 48 hours schedule is insufficient.

Fig 10 also shows simulations of 0.1 mg/g Moxifoxacin treatment every 12 hours and every 48 hours, starting 24 hours after infection. The denser treatment eliminates the bacteria faster than the 48 hours schedule, but still, both therapy schedules are predicted to result in cure.

**Fig 11:**
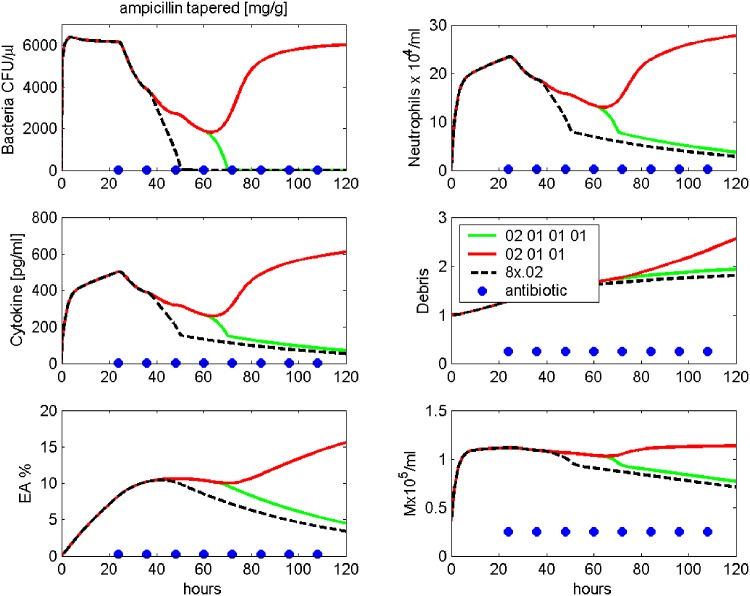
Prediction: Dose reduction in antibiotic treatment. Treatment with modified Ampicillin schedule after infection with 5 ⋅ 10^6^ Streptococcus pneumoniae are simulated. Time series of pneumococcal population, neutrophils, IL-6, debris, EU, EA and macrophages in BALF are presented. Therapy starts at 24 h after infection with 0.02 mg/g. Then, dose is reduced to 0.01 mg/g given every 12 hours. Ampicillin treatment is stopped after 48 h (red line) or 60 h (green line). The black dashed line shows the standard treatment with Ampicillin 0.02 mg/g every 12 hours, stopped after 108 hours.

In Fig 11, another scenario is modelled. Here we gradually reduced the dose of Ampicillin treatment during the course of the therapy. In more detail, therapy starts at *t*_1_ = 24 hours after infection with the standard 0.02 mg/g. Dose is reduced to 0.01 mg/g at 36 hours and 48 hours. At 60 hours, about 2000 CFU/*μl* are left. Stopping treatment here results in relapse, while an additional treatment with 0.01 mg/g results in cure. However, elimination of bacteria takes considerably longer compared to standard therapy, where 0.02 mg/g Ampicillin is given every 12 hours until 108 hours after infection.

## Discussion

In the present paper, we propose a biomathematical model of systemic lung infection in mice caused by *streptococcus pneumoniae*. The model is based on differential equations of the dynamics of pneumococci in the lung, immune cell response, inflammatory cytokines represented by IL-6 and epithelial barrier damage. We also include antibiotic treatment of the disease incorporating the two antibiotic drugs Ampicillin and Moxifloxacin for the first time. The system is parametrized on the basis of mice experimental data. It is our intention to keep the model as simple as possible in order to keep a balance between biological complexity of the immune response and the identifiability of parameters on the basis of the limited amount of available experimental data. We obtained a good agreement of model and data showing that our simple assumptions regarding action of antibiotic treatments are sufficient to explain the data. After establishing the model, we demonstrate how it can be used to make relevant and testable predictions regarding new therapy options opening a way to validate the model. We predict for example that cure can be achieved with lower doses of antibiotics as those applied in the current setting. However, further refinements of the model are required to incorporate more complex regulatory mechanisms of immune response, and especially, to address the early phase of infection.

There is a large amount of literature regarding different modelling aspects of the immune system during lung infection. Proposed models can be categorized by the pathogen responsible for the lung infection. For example a number of models have been proposed to describe immune dynamics during viral infection by e.g. influenza A (see [19] for an overview of models). Models of fungal infection by *Aspergillus fumigatus* have been proposed by Pollmächer and Figge [20, 21]. Here we focus on infections with *streptococcus pneumoniae*. Again some models were already proposed for this situation. Guo et al. [22] presented a simple model of pneumonia under immuno-suppression. The model of Mochau et al. [23] included some more features, namely neutrophil response and transitions of pneumococci between lung and circulating blood. Smith et al. [4] proposed an elaborated model considering alveolar macrophages, immigrated neutrophils and immigrated monocytes differentiating to macrophages as three important lines of immune cell response. The model also considers affection of the endothelial barrier. Shreshta et al. [24] combined this model with a model of influenza infection in order to study their interaction.

Our model mainly relies on the work of Smith et al. [4]. However, a number of adaptations were necessary to obtain the desired model behaviour and to explain our data of lung infection. Moreover, we included the effect of antibiotic treatment which was not considered in Smith et al. In more detail, we do not distinguish between alveolar macrophages and monocyte derived macrophages when modelling macrophage induced killing of pneumococci. We also assumed a saturated kill kinetic of pneumococci in contrast to Smith et al. For the unaffected endothelial cells, we assumed a production term which has the advantage that the system returns to steady-state after successfully fighting the infection. While the neutrophil and the debris compartment are modelled in analogy to Smith et al., we modified the equation of macrophages. The delay term proposed by Smith was dropped and an initial condition different from zero was chosen representing alveolar macrophages. In summary, our adaptations did not increase the complexity of the model. Antibiotic treatment was added to the model assuming a two-compartment injection model and a kill term proportional to the product of the number of pneumococci and the drug concentration. In summary, it turned out that the assumptions made by Smith *et al*. regarding major players and mechanisms of the interactions of disease and immune system proved to be reasonable to explain our data. Only a few adaptations were necessary regarding the concrete realization of equations.

We showed that our model has a number of properties desirable for biological plausibility. At first, we proved that the system has a unique and stable pneumonia-free steady state. Moreover, we showed that there is a bifurcation regarding the initial number of pneumococci resulting in either cure or disseminated disease. Finally, derived model parameters suggest that the (alveolar) macrophages are responsible for early elimination of the disease.

Parameters of our model were either kept constant (e.g. values proposed by Smith et al.) or were estimated by fitting the predictions of the model to available experimental data. Experimental data comprised time-series of measurements in infected mice with or without antibiotic treatment [9]. Experimental readouts were mapped to appropriate model compartments. However, for some model compartments only proxy measurements are available. As example, endothelial permeability was estimated by the ratio of BALF and murine serum albumin and was mapped to the model compartment of affected endothelial cells. The dynamics of the debris compartment was compared with a semiquantitatively assessed histological score of the lung tissue. One has to acknowledge here that debris is not coupled with any other compartment of our system, and hence, was only considered to explain the data. Since we used the same model for debris than in Smith *et al*., data could in principle be used to justify the equation.

Sensitivity analysis of model parameters showed that not all parameters could be identified with high certainty. We expect that this issue could be improved by better data, especially close meshed time series in the early phase of the infection which are still lacking. We plan to complete our data base regarding this issue in the near future especially with respect to data from the early phase of infection which is unsufficiently covered at the moment to address quick immune responses. Identified parameter sets resulted in a good agreement of model prediction and data. However, acknowledging the above mentioned issues, our parameter set is preliminary requiring validation by additional experiments.

There are several ways to improve and further extent our model: At first, new therapeutic interventions such as other antibiotic drugs could be included. Complexity of the model could be improved if other readouts of the experiments are available such as comprehensive dynamics of relevant cytokines. Qualitatively the model could be refined by adding a systemic response model. This is not considered so far and could be a reason why the fit of data of untreated mice which might become septic is less optimal than that of treated mice which were cured, probably not reaching septic states. Several models of systemic infection and extrapulmonary organ involvement were proposed in the literature and could be combined with our model in the longer perspective (for example [8, 25–28]). However, we believe that data base must be improved first to construct such comprehensive models containing numerous unknown parameters and mechanisms. Therefore, both, model equations and parameters must be considered as preliminary.

We conclude that we established a biomathematical model of pneumonia explaining qualitative phenomena as well as quantitative time series data of infected mice with and without antibiotic treatment. We aim at improving the model on the basis of additional mice experiments currently performed considering early time points and novel therapeutic options. We also aim at translating our mouse model to the human situation.

## Supporting Information

S1 Appendix The supplement file S1_Appendix.pdf contains numerically obtained values for the of the chronic infection steady-state and corresponding stability analysis. (PDF)
