# Supplementary material for: A Biomathematical Model of Pneumococcal Lung Infection and Antibiotic Treatment in Mice
Source: PLoS One. 2016 May 19;11(5):e0156047. doi: 10.1371/journal.pone.0156047 (PMC4873198; doi:10.1371/journal.pone.0156047)
Supplement: S1 Appendix — (PDF) [file pone.0156047.s001.pdf]

A Biomathematical Model of Pneumococcal Lung Infection and Antibiotic Treatment in Mice: Supplement Material

Sibylle Schirm[1], Peter Ahnert[1], Sandra Wienhold[2], Holger Mueller-Redetzky[2], Geraldine Nouailles-Kursar[2], Markus Loeffler[1], Martin Witzenrath[2], Markus Scholz[1,3*]

1 Institute for Medical Informatics, Statistics and Epidemiology, University of Leipzig, Leipzig, Germany
2 Department of Internal Medicine/Infectious Diseases and Respiratory Medicine Charité – Universitätsmedizin Berlin, Berlin, Germany
3 LIFE Research Center of Civilization Diseases, University of Leipzig, Leipzig, Germany

* E-mail: markus.scholz@imise.uni-leipzig.de

Stability of the Chronic Infection Steady-State

The infection-free steady-state is not globally stable. Starting e.g. with an initial bacterial load of $5 \cdot 10^6$, the system results in persistent infection. For this equilibrium, an analytical solution is not available. A numerical approximation results in $P_1 = 6.172361 \cdot 10^3$, $EU_1 = 25.99491$, $EA_1 = 7.159392$, $N_1 = 2.266410 \cdot 10^5$, $M_1 = 1.112171 \cdot 10^5$, and $C_1 = 4.832019 \cdot 10^2$. Fig 1 shows this steady state which can be interpreted as chronic infection. It turns out that the steady state is also locally stable.

Proof. The Jacobian for the steady-state reads as follows

$$\mathbf{J_1} =$$

$$\begin{pmatrix} B & 0 & 0 & -\frac{k_{\mathrm{PN}} \cdot P_1}{n+P_1} & -\frac{k_{\mathrm{PM}} \cdot P_1}{n+P_1} & 0 \\ -k_{\mathrm{EUP}} \cdot EU_1 & -\frac{k_{\mathrm{EU}}}{EU_0} - k_{\mathrm{EUP}} \cdot P_1 & 0 & 0 & 0 & 0 \\ k_{\mathrm{EA}} \cdot EU_1 & k_E A \cdot P_1 & -d_E 0 & 0 & 0 & \\ -d_{\mathrm{NP}} \cdot N_1 & 0 & 0 & -\frac{k_{\mathrm{NC}} \cdot C_1}{N_{\max}} - d_{\mathrm{NP}} \cdot P_1 - d_N & 0 & k_{\mathrm{NC}} \cdot (1 - \frac{N_1}{N_{\max}}) \\ 0 & 0 & 0 & 0 & -d_M & \frac{k_{\mathrm{MAC}} \cdot k_M^2}{(C_1 + k_M)^2} \\ k_{\mathrm{CMA}} \cdot M_1 & 0 & k_{\mathrm{CEA}} & 0 & k_{\mathrm{CMA}} \cdot P_1 & -d_C \end{pmatrix}$$

where $B = k_P \cdot \left(1 - \frac{2 \cdot P_1}{P_{\max}}\right) - \frac{(k_{\mathrm{PN}} \cdot N_1 + k_{\mathrm{PM}} \cdot M_1) \cdot n}{(n+P_1)^2}$ Numerically determined eigenvalues of $\mathbf{J_1}$ are $-2.0090 \cdot 10^2$, -2.8158, $-5.3898 \cdot 10^{-1} + 7.8228 \cdot 10^{-2} i$, $-5.3898 \cdot 10^{-1} - 7.8228 \cdot 10^{-2} i$, $-1.5513 \cdot 10^{-2}$, $-6.1320 \cdot 10^{-3}$. Since all eigenvalues have strictly negative real part, the steady-state is locally asymptotically stable.

□

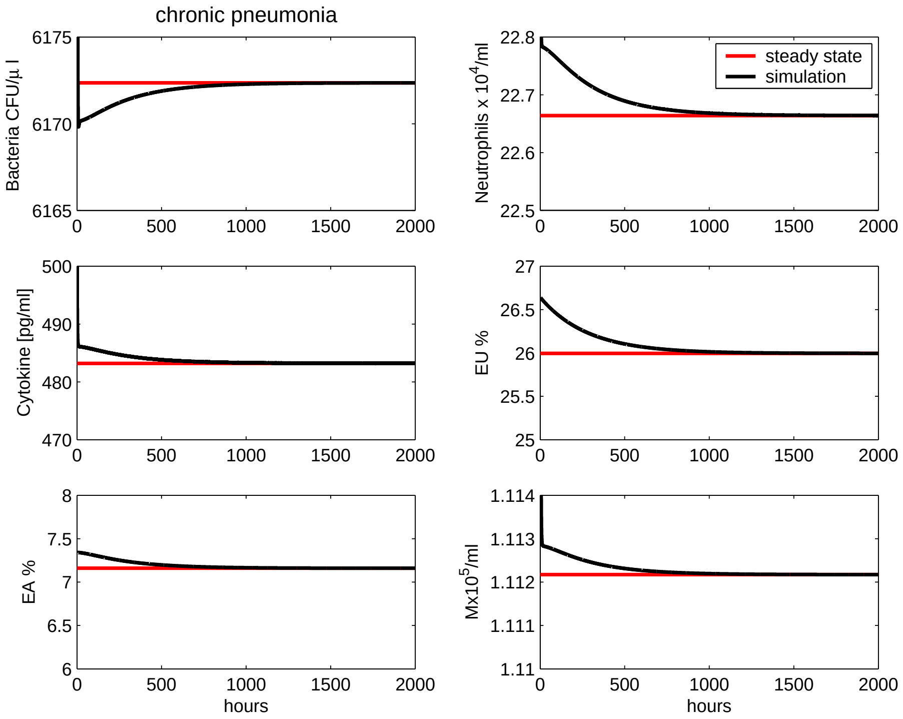

Fig 1. Chronic Infection Steady-State. Time series of pneumococcal population, neutrophils, IL-6, EU, EA and macrophages in BALF are presented. The simulaton starts with the initial steady state values P_1, EU_1, EA_1, N_1, M_1, and C_1, changed by +2.5 % (black curves), and then, returns to it (red lines).
